# Sensory Quality, Volatile Compounds, and Physical Properties of Sheep’s Milk Cheese with Herbs (*Allium ursinum* L.)

**DOI:** 10.3390/molecules29245999

**Published:** 2024-12-19

**Authors:** Agnieszka Pluta-Kubica, Dorota Najgebauer-Lejko, Jacek Domagała, Jana Lakatošová, Marek Šnirc, Jozef Golian

**Affiliations:** 1 Department of Animal Product Processing, Faculty of Food Technology, University of Agriculture in Krakow, Balicka 122, 30-149 Krakow, Poland; agnieszka.pluta-kubica@urk.edu.pl (A.P.-K.); jacek.domagala@urk.edu.pl (J.D.); 2 AgroBioTech Research Centre, Slovak University of Agriculture in Nitra, Tr. A. Hlinku 2, 949 76 Nitra, Slovakia; jana.lakatosova@uniag.sk; 3 Institute of Food Sciences, Faculty of Biotechnology and Food Science, Slovak University of Agriculture in Nitra, Tr. A. Hlinku 2, 949 76 Nitra, Slovakia; marek.snirc@uniag.sk (M.Š.); jozef.golian@uniag.sk (J.G.)

**Keywords:** soft cheese, wild garlic, flavor, volatiles, color, texture

## Abstract

The aim of this study was to investigate the effect of the addition of wild garlic leaves on the sensory quality, volatiles, color, and texture of sheep milk soft rennet-curd cheese. The sensory evaluation of color, appearance, texture, odor, and taste was performed using a 5-point scale. The intensity of selected taste and odor discriminants was also assessed. Volatiles were analyzed by the GC-MS method. Color and textural characteristics were determined instrumentally. The wild garlic addition had no effect on the sensory characteristics of the cheese (*p* > 0.05). However, cheese with herbs exhibited a less intensive sour odor (*p* ≤ 0.05), sheep’s milk odor, and taste (*p* ≤ 0.01). (E)-7-methyl-4-decene, dichloroacetic acid undecyl ester, and 3,5-dimethyl-octane, described as creamy, acetic, and acid pungent in smell, were not detected in the cheese with wild garlic while they were present in the natural one. Moreover, herbal cheese was more piquant (*p* ≤ 0.01). PCA showed that the differences in volatiles resulted both from the use of wild garlic and the time of storage. Herbal addition affected almost all color characteristics, except for the hue angle (h), but caused an increase only in hardness and chewiness. In conclusion, wild garlic leaves can be recommended as an additive in the production of soft sheep’s milk rennet-curd cheese.

## 1. Introduction

Fresh, soft rennet-curd cheese can be manufactured from the milk of various animal species, e.g., sheep. Sheep’s milk is an excellent, well-known matrix for cheese production [1]. The milk intended for fresh soft cheese production can be either raw or pasteurized. Its manufacture involves the utilization of lactic acid bacteria (LAB) with mixed-strain mesophilic starter cultures and an enzyme called rennet [2]. Herbs are a common additive used in the production of soft cheeses. The aim is to enrich the cheese flavor and increase the variety of the assortment. It is often related to the tradition of the production region. Herbs contribute many aroma-active compounds. Moreover, their presence in cheese promotes fat hydrolysis during ripening, causing the release of higher levels of free fatty acids [3]. Therefore, the addition of herbs to the cheese matrix changes the profile of volatile compounds, both indirectly and directly.

Sheep’s milk is mainly used for the manufacture of fine cheese varieties, yogurt, and whey cheeses. It has a beneficial proximate composition and vitamin and mineral content but is not as popular as bovine milk due to its seasonality [1]. Ovine milk, when compared to bovine or caprine milk, is richer in nutrients. In particular, it is characterized by high protein and casein contents, which makes it an excellent raw material for cheese production. About 220,000 sheep are bred in Poland, mainly in the mountain areas [4]. Unfortunately, the amount of collected ewes’ milk is relatively small and equals about 100 tons per year. Approximately 70% of it is utilized for cheese production. Among the cheeses produced from ovine milk in Poland, the most popular are Bryndza Podhalańska, Oscypek, and Redykolka (Protected Designation of Origin Cheeses). In France, the milk of the Lacaune sheep breed is used for the production of Roquefort cheese [5]. In general, ewes milk cheese is characterized by many aroma-active compounds with odor descriptors such as animal, sour, fatty, burnt, salty, fermented, foot-like, and sweaty [6].

Wild garlic (*Allium ursinum* L.) is used in local cuisine in Eastern European countries, as well as in the Czech Republic, Poland, Germany, and Turkey. Wild garlic leaves are most commonly used by consumers. However, the bulbs and flowers of this plant are also edible. Its leaves are used in the manufacture of local rennet-curd cheeses, e.g., in Turkey and Poland [7].

It has been scientifically proven that wild garlic, due to its bioactive compounds, can be used in the prevention of cardiovascular diseases and cancers, as well as being characterized by a strong bactericidal effect [8]. Moreover, wild garlic is a rich source of sulfur and phenolic compounds of high antioxidant activity. Recently, wild garlic extract has been successfully incorporated into polylactide films for food packaging [9]. Furthermore, attempts have been made to develop environmentally sustainable processes for making time-stable *Allium ursinum* extracts for food applications, which aim to take advantage of all-powerful bioactive compounds present in this plant [10].

Wild garlic leaves contain numerous bioactive compounds, including sulfur compounds, such as S-alk(en)yl-L-cysteine, sulfoxides (methiin, alliin, isoalliin, ethiin, propiin), thiosulfinates (allicin, methyl-allyl thiosulfinate, dimethyl thiosulfinate), and (poly)sulfides. They are also rich in polyphenolic compounds, e.g., gallic and ferulic acid, kaempferol derivatives, as well as carotenoids, and the antioxidant enzymes catalase and glutathione peroxidase [8,11,12]. These compounds, when added to cheese, may have a beneficial effect on the human body as well as on the physicochemical and sensory properties of the cheese.

The investigation of the influence of wild garlic leaves on the properties of two kinds of cow milk cheeses, herby pickled cheese and unripened soft rennet-curd cheese revealed their effect on color, which was determined using the CIELAB system. Differences were also found in sensory characteristics between the control cheese and cheeses with herbal additions [3,13].

It has been proven that consuming wild garlic has a beneficial effect on the human body [8]. In addition to health-promoting properties, when added to cheese, wild garlic can also provide unique sensory characteristics, such as taste and aroma. Therefore, it was chosen as an ingredient in the cheese investigated in this study. We hypothesized that the addition of wild garlic leaves could have a positive influence on the quality of soft sheep’s milk rennet-curd cheese. Therefore, the aim of the study was to investigate the effect of the addition of wild garlic leaves on the sensory quality, volatile compounds, color, and texture of sheep’s milk soft rennet-curd cheese.

## 2. Results and Discussion

### 2.1. Chemical Composition, Acidity and Water Activity of Cheese

The basic chemical composition, acidity, and water activity of both analyzed sheep cheese types are presented in Table 1. Herbal additions had almost no effect on the chemical composition of the cheese, except for water content. The amount of water was lower in the samples with wild garlic than in the control (*p* ≤ 0.05). Similarly, a higher dry matter content compared to the control was reported in double cream cheese supplemented with *Allium roseum* [14] as well as in our previous studies regarding soft cow’s milk cheese with *Allium ursinum* [13].

Generally, the average pH of the control was significantly higher than that of cheese with herbs (*p* ≤ 0.05). However, the observed difference, although statistically significant, was somewhat small. The mean pH of the control cheese was 4.70, while the pH of the herbal cheeses was, on average, 4.68. On the other hand, plain cheese was subjected to a greater increase in pH during storage compared to herbal cheese (Table 1).

The water content in the cheese was stable during storage. However, pH and water activity significantly increased after two weeks of refrigeration (*p* ≤ 0.05). It is possible that the increase in pH was caused by proteolytic changes during storage, e.g., a higher water-soluble nitrogen content after ripening is related to higher pH values [15].

Differences in the water content between the control cheese and the cheese with wild garlic should be taken into account during production to ensure that the cheese’s dry matter content is still in accordance with the compositional standards for soft cheese. The percentage of moisture on a fat-free basis in soft cheese should be higher than 67 [16].

### 2.2. Sensory Quality of Cheese

The results of sensory quality evaluation are shown in Table 2. The addition of wild garlic had no significant influence on the cheese’s sensory features assessed using the 5-point scale. Regarding the time effect, it was significant in terms of color (*p* ≤ 0.05), odor (*p* ≤ 0.01), and overall quality (*p* ≤ 0.05). The storage time was associated with a decrease in the assessment of the aforementioned quality characteristics. No significant interactions were found between the effects. It has been previously reported that sensorial properties of herby-pickled cheese, such as appearance, color, and flavor, worsen between 2 and 30 days of storage [3].

The intensity of the discriminators of the odor and taste assessment revealed that cheese samples with wild garlic were characterized by a less intensive sour odor (*p* ≤ 0.05), sheep’s milk odor and taste (*p* ≤ 0.01). Similarly, fermented milk with wild garlic added was found to exhibit a less intensive sour smell and milky/creamy taste [17]. Moreover, herbal cheese was more piquant (*p* ≤ 0.01). Likewise, a pungent and spicy taste was more pronounced in the double cream cheese samples with *Allium roseum* in comparison to the plain ones [14]. Soft sheep cheese with wild garlic also had a more intensive foreign taste (*p* ≤ 0.01). However, its average perceptions were below 0.50. Obviously, wild garlic taste and odor were imperceptible in natural cheese samples and intense in those with wild garlic added (*p* ≤ 0.001). Interestingly, the intensity of the sour odor was lower during storage (*p* ≤ 0.05). However, the storage time was associated with a stronger perception of rancid odor (*p* ≤ 0.01) and bitter taste (*p* ≤ 0.05). Nevertheless, the average results of these features were lower than 1.00. Therefore, their perception was not intensive.

The results obtained show that wild garlic provides unique sensory characteristics, enriching cheese’s odor and taste. This may have a positive influence on consumer acceptance. However, to determine this, additional research should be conducted in the future using consumer methods to assess product acceptability. An example of such a method would be the use of a hedonic scale [18].

### 2.3. Volatile Compounds in Cheese

Volatile compounds in fresh and stored sheep milk cheese samples determined by GC-MS are listed with their sensory descriptors in Table 3. The presence of the following compounds was related to the addition of wild garlic: 2-hexyl-1-dodecanol, 2-octyl-1-dodecanol, and 1,1,2,2-tetrachloroethane were detected in fresh herbal cheese; 1-pentadecene, and 1-tetradecanol were found in stored cheeses; and (E)-2-decenal was detected regardless of storage time. Tetradecyl alcohol (1-tetradecanol) was previously identified in the aqueous extract of *Allium chinense* G. Don. [19], while 2-octyl-1-dodecanol was detected in *Allium prezewalskianum* regel [20].

The obtained results also enabled the indication of volatile compounds present only in the natural (plain) cheese samples. Six compounds (2-methyl-1-decanol, 2,4-dimethyl-1-decene, 1-dodecanol, (E)-3-octadecene, 3,5-dimethyl-octane, and 3,8-dimethyl-undecane) were detected only in fresh samples without herbal addition, and another six chemicals (1-phenyl-1,2-propanediol, (E)-7-methyl-4-decene, carbonic acid eicosyl vinyl ester, dichloroacetic acid undecyl ester, 2,6-dimethyl-nonane, and 3,3-dimethyl-octane) were found only after natural cheese storage while tetrachloroethylene was characteristic of both fresh and stored samples.

Only two compounds, 1-tetradecene and 3-ethyl-3-methylheptane, were detected both in N and H fresh cheese samples but were not present anymore after their storage. Moreover, 1-butyl-2-propylcyclopentane and 2-(dodecyloxy)-ethanol were revealed only in both N- and H-stored cheese samples.

**Figure 1:**
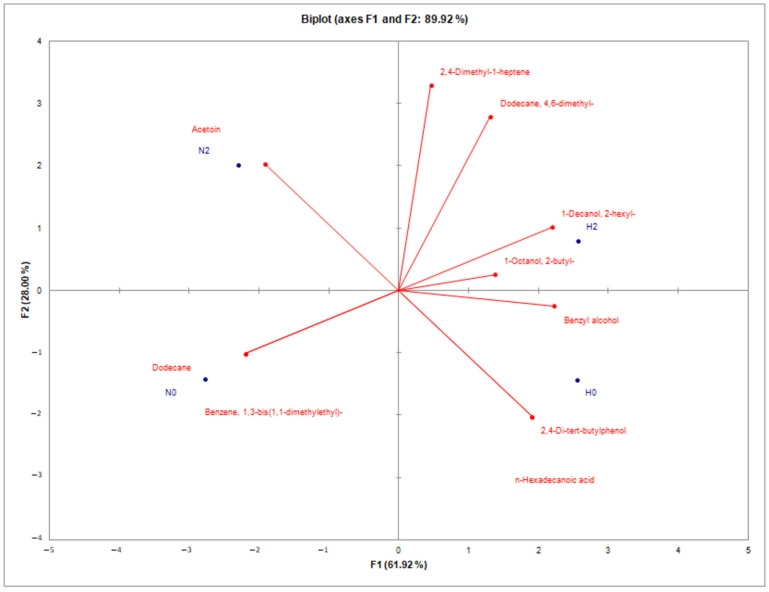
PCA of the volatile compounds of sheep cheeses. Abbreviations: N—natural; H—with wild garlic; 0—fresh; 2—stored for two weeks.

Principal component analysis (PCA) (Figure 1) revealed that the two factors (F1 and F2) explained a total of 89.92% of the overall variability in the data. F1, with an eigenvalue of 6.19, accounted for nearly 62% of the total variability, while F2, with an eigenvalue of 2.8, accounted for 28% of the total variability. The most significant variables for F1 were 2-hexyl-1-decanol, benzyl alcohol, 1,3-bis(1,1-dimethylethyl)-benzene, and dodecane. For F2, the most important variables were 2,4-dimethyl-1-heptene and 4,6-dimethyl-dodecane. Sample N2 was characterized by a high content of acetoin. It is a common compound in cheese, the presence of which is associated with the metabolism of lactic acid bacteria [21]. In contrast, sample N0 was characterized by high levels of dodecane and 1,3-bis(1,1-dimethylethyl)-benzene. The dodecane content decreases during storage; therefore, its high level was expected in the fresh N0 samples [22]. The 2-hexyl-1-decanol, 2-butyl-1-octanol, and benzyl alcohol contents were typical of H2. H0 was characterized by amounts of 2,4-di-tert-butylphenol and n-hexadecanoic acid. The last-mentioned compound was previously determined in Allium ursinum samples harvested in Poland [11].

The sensory analysis revealed that storage time was associated with a stronger perception of a rancid odor (Table 2). Compounds such as 1-phenyl-1,2-propanediol, 1-tetradecanol, (E)-7-methyl-4-decene, carbonic acid eicosyl vinyl ester, dichloroacetic acid undecyl ester, 2,6-dimethyl-nonane, and 3,3-dimethyl-octane were detected only after storage. The odor of some of them is described as pungent, fatty, or acetic. Therefore, their smell could have caused the perception of rancid odors. Moreover, the cheese with wild garlic was characterized by less intensive sour and sheep’s milk odor. The cause of these differences could have been the fact that (E)-7-methyl-4-decene, dichloroacetic acid undecyl ester, and 3,5-dimethyl-octane, described as creamy, acetic, and acid pungent in smell, were not detected in the cheese samples with wild garlic while they were present in the natural samples.

Similarly to the results of the sensory analysis, the effects of determining volatile compounds in cheeses showed that wild garlic addition affects them qualitatively. A few compounds exhibiting creamy, acetic, and acid pungent odor, although detected in the natural cheese samples, were not present in the ones with wild garlic. This may positively affect the consumer’s acceptance of the cheese’s smell. However, additional research should be conducted to determine this.

### 2.4. Physical Properties of Cheese

Color characteristics and textural properties of sheep cheeses are shown in Table 4. Herbal addition affected almost all color characteristics except for the hue angle (h). The application of wild garlic caused a significant lowering of L* values. Moreover, herbal cheese had higher color saturation intensity (C*) and was more greenish and yellowish than the control cheese. The same trends were found in studies on natural sheep kefirs and kefirs with wild garlic [8]. Similarly, fermented milk with wild garlic added [17], and herby-pickled cheese [3] were found to exhibit lower lightness and more intensive green color in comparison to the control samples.

Only the lightness of the cheese was stable during storage. The samples stored were significantly more greenish and yellowish than the fresh samples. Moreover, h and C* became lower after storage. Herby-pickled cheese was also found to become more yellowish during storage; however, it also seemed less greenish and brighter [3].

The calculation of ΔE demonstrated that the storage triggered a minor color change in N and H cheeses, which could be detected by the human eye (ΔE equaled 1.35 and 1.02, respectively).

The herbal addition caused an increase in hardness and chewiness. Most likely, this was caused by the lower water content in the samples with wild garlic than in the control (Table 1). Other textural parameters were not significantly affected. The addition of wild garlic also caused an increase in hardness and chewiness in sheep milk kefirs [8].

Hardness and adhesiveness were stable during storage. However, springiness, cohesiveness, and chewiness became lower after 2 weeks of refrigeration. The lowering of springiness and cohesiveness values during storage was probably caused by the increase in pH. Moreover, differences in chewiness values were the result of the method of calculation—multiplying hardness by springiness and cohesiveness [23].

All changes in L*, a*, b*, and C* values, as well as hardness and chewiness levels, had no adverse effect on the sensory assessment of the color, appearance, or texture of the cheese with herbs. The quality of these features was assessed as very good (Table 2). Therefore, the changes in instrumental color and texture characteristics will probably not affect consumer acceptance.

## 3. Materials and Methods

### 3.1. Materials

Raw ovine milk (from the Lacaune breed) was obtained in the summer (from July to September) from a farm located in Kłomnice near Częstochowa (Poland). It was transported under refrigerated conditions to the laboratory and processed immediately. Wild garlic leaves were harvested in Ropa. All the details regarding the plant additive were highlighted in a previous manuscript [13]. Briefly, a convection drying method was applied using a ProfiLine-type chamber dryer with airflow parallel to the sieves (Hendi, The Netherlands). The drying temperature was 40 °C, and the time was about 40 h to achieve the final humidity of the leaves equal to 10%.

Soft cheese was prepared on a laboratory scale and in two independent batches at the Faculty of Food Technology, University of Agriculture in Krakow (Poland). A previously described method of production was used with minor modifications [13]. The milk was not standardized to fat content before pasteurization, and the cheese was produced using a cheese kettle mini SKM 50 (PLEVNIK d.o.o., Dobrova, Slovenia). According to the cited publication, the same temperature and time were applied during all production steps. Anhydrous calcium chloride (0.2 g/kg of the vat milk), mesophilic mixed strain starter culture CHN-19 (*Lactococcus lactis* subsp. *cremoris*, *Lactococcus lactis* subsp. *lactis*, *Leuconostoc mesenteroides* subsp. *cremoris* and *Lactococcus lactis* subsp. *diacetylactis*, Chr. Hansen, Hørsholm, Denmark), and microbial rennet with an activity of 2200 IMCU/g (Fromase 2200TL, Specialities, Heerlen, Denmark) were added. The curd was separated into two groups prior to molding. Half of it was intended for the production of natural cheese (N). The rest of the curd was gently mixed with air-dried and chopped wild garlic leaves (5 g/kg of the curd) (H). The cheeses were drained, brined, dripped, individually packed in LLDPE film, and stored for 2 weeks at 4 °C.

### 3.2. Chemical Composition, Acidity, and Water Activity Evaluation

The content of water and fat in cheese were determined in accordance with ISO 5534:2004 [24] and ISO 3433:2008 [25], respectively. The remaining dry matter components, i.e., protein, ash, and NaCl, were analyzed according to AOAC [26]. A pH meter (CP-411, Elmetron, Zabrze, Poland) was used to assess the acidity of the cheese. The water activity of cheese was measured using LabMaster-aw (Novasina AG, Lachen, Switzerland) [27]. The chemical composition, pH, and water activity of cheese were evaluated on the day following brining. Additionally, the water content, pH, and water activity were determined at the end of the storage period. All analyses were performed in triplicate.

### 3.3. Sensory Quality Assessment

The sensory evaluation of cheese samples was performed according to two methods. First, color, external, and cross-sectional appearance, as well as texture, odor, and taste, were evaluated using a 5-point scale (1—“bad quality”, 2—“insufficient quality”, 3—“satisfactory quality”, 4—“good quality”, and 5—“very good quality”). The following indices of importance, 0.15, 0.20, 0.15, 0.25 and 0.25, respectively, were used to evaluate the overall quality [28]. Afterwards, an assessment of the intensity of taste and odor discriminants (from 0—imperceptible to 5—very intense) was performed using the profiling method scale [29]. The following discriminants were taken into account: sheep milk odor, sour odor, wild garlic odor, desirable odor, rancid odor, foreign odor, sheep milk taste, sour taste, bitter taste, piquant taste, salty taste, wild garlic taste, desirable taste, rancid taste, and foreign taste.

Each type of cheese was evaluated by 10 trained panelists (n = 80; 2 kinds of cheese × 2 storage periods × 2 series of production × 10 panelists). A sensory quality assessment was performed the day after the brining process and after 2 weeks of storage. It took about 10 min per evaluator. Potable water was available to the panelists during the analysis.

### 3.4. Volatile Compounds Analysis

The extraction of volatile compounds was performed with modifications [30] as follows. An amount of 2 g of the sample was extracted with dichloromethane for HPLC ≥ 99.8% (5 cm^3^; Sigma-Aldrich Merck KGaA, Darmstadt, Germany) by a shaker (Unimax 2010, Heidolph, Schwabach, Germany) at a laboratory temperature (18 °C ± 0.1 °C) for 3 h and then filtered through a syringe PVDF filter (0.45 µm × 13 mm; Chromservis, Bratislava, Slovakia). Afterwards, 1 cm^3^ of the sample extracts were stored in 2 cm^3^ vials (Agilent Technologies Inc., Santa Clara, CA, USA).

The analysis of the extract was carried out using gas chromatography-mass spectrometry (GC-MS) (GC 7890B coupled by MSD 5977A; Agilent Technologies Inc.) equipped with CombiPal autosampler CTC120 (CTC Analytics AG, Zwingen, Switzerland). A column HP-5ms (30 m × 0.25 mm × 0.25 µm; Agilent Technologies Inc.) was used. One microliter of the sample extract was injected into the inlet and operated in a split mode 10:1 at 250 °C. The oven temperature program started at 40 °C. The temperature was held for 3 min, then increased to 250 °C at 3 °C/min, and was then held again for 10 min. Helium was used as a carrier gas at a constant flow (1.2 cm^3^/min). The mass detector parameters were as follows: ionization energy of filament: 70 eV; transfer line temperature: 250 °C; MS source temperature: 230 °C; and quadrupole temperature: 150 °C. The mass spectrometer was programmed under electron impact (EI) in a full-scan mode at m/z 40–450 with a frequency of 1.8 scans/s. Each sample was measured in triplicate (n = 24; 2 kinds of cheese × 2 storage periods × 2 series of production × 3 repetitions).

The compound identification was carried out by comparing mass spectra (over 80% match) with a commercial database NIST library 2017 (National Institute of Standards and Technology, Gaithersburg, MD, USA) and Wiley library, and retention times of the reference mixture standard of n-alkanes (11 components, Restek Corporation, Bellefonte, PA, USA). The relative percentage (%) of the determined volatile compounds was calculated by dividing the individual peak area by the total area of all the peaks.

### 3.5. Physical Properties Analysis

The cheese color was evaluated as previously described [31]. A Konica Minolta CM-3500d spectrophotometer in the reflectance mode under the illuminant D65/10° (Konica Minolta Sensing Inc., Osaka, Japan) was utilized. L*—lightness (from 0—black to 100—white); a* coordinate—from greenness (negative values) to redness (positive values); and b* coordinate—from blueness (negative values) to yellowness (positive values) were determined in the CIE L* a* b* system. Moreover, h°—hue angle and C*—chroma (saturation) were calculated. Every cheese sample was cut into 6 cubes with a side length of 2 cm. Afterwards, the temperature of the cubes was adjusted to 20 °C, and their color was determined twice (n = 96; 2 kinds of cheese × 2 storage periods × 2 series of production × 6 cubes × 2 repetitions).

Additionally, the total color difference value (ΔE) between fresh and stored samples was calculated (Equation (1)).

(1) $$\Delta E=\sqrt{{\left(∆L^{*}\right)}^{2}+{\left(∆a^{*}\right)}^{2}+{\left(∆b^{*}\right)}^{2}}$$

where ΔL*, Δa*, and Δb* are the differences between the values of the respective color characteristics of the cheese types compared. ΔE can be interpreted as follows: ΔE > 3 means that color differences could be easily detected by the human eye; values of ΔE in the range of 1–3 mean that minor color differences could be seen; and ΔE lower than 1 proves that color differences could not be perceived by the human eye [32].

Instrumental texture profile analysis (TPA) was performed exactly as previously described [33] using the Universal Texture Analyzer TA-XTPlus (Stable Micro Systems, Surrey, UK), controlled by a computer. Every cheese sample was prepared similarly to the color measurement (n = 48; 2 kinds of cheese × 2 storage periods × 2 series of production × 6 cubes). The obtained diagrams of force dependence on time were analyzed using Texture Expert for Windows v. 1.05 (Stable Micro Systems, Surrey, UK). The Fracture TPA algorithm allowed the calculation of the hardness, adhesiveness, springiness, cohesiveness, and chewiness of cheese samples.

### 3.6. Statistical Analysis

Analyzes were performed in triplicate unless otherwise stated. The obtained results, except volatile compound determination, were statistically analyzed using Statistica version 13.3 (TIBCO Software Inc., Palo Alto, CA, USA). Means and standard deviations were calculated. A two-way ANOVA was applied to estimate the effect of both independent variables, i.e., wild garlic addition and storage time. The effect of herbal addition on the concentration of fat, protein, ash, and NaCl in cheese samples was estimated using the t-test. The null hypothesis was discarded for *p* ≤ 0.05 in all statistical analyses.

All the data obtained during the analysis of volatiles were analyzed by descriptive statistics for the arithmetic average and standard deviation. Then, all the variables were tested for normality. According to the Shapiro–Wilk test and the Kolmogorov–Smirnov test, all the tested variables did not follow the Gaussian distribution. The ten most numerous volatile compounds (≥5% AREA), characteristic of each sample, were used for PCA. Principal component analysis (Spearman type) was used to find a pattern of similarity between the observations and the variables by displaying them as points on a map. Descriptive statistics, normality tests, and the PCA were performed using the MS Excel and XLSTAT package programs [34].

## 4. Conclusions

To the best of our knowledge, soft rennet-curd sheep milk cheese supplemented with wild garlic leaves has been investigated for the first time in this study. Our research demonstrated that the addition of wild garlic and the storage duration significantly influence various ovine milk cheese characteristics. Cheese samples with herbs exhibited a less intensive sour odor (*p* ≤ 0.05), sheep’s milk odor, and taste (*p* ≤ 0.01). (E)-7-methyl-4-decene, dichloroacetic acid undecyl ester, and 3,5-dimethyl-octane, described as creamy, acetic, and acid pungent in smell, were not detected in the cheese with wild garlic while they were present in the natural cheese. Moreover, herbal cheese was more piquant (*p* ≤ 0.01). Interestingly, the intensity of the sour taste decreased during storage (*p* ≤ 0.05). PCA showed that the differences in volatiles resulted both from the use of wild garlic and the time of storage. Herbal addition affected almost all color characteristics, except for the hue angle (h), but caused an increase only in hardness and chewiness. The calculation of ΔE demonstrated that the storage triggered a minor color change in N and H cheeses, which could be detected by the human eye. All aforementioned changes had no adverse effect on the sensory assessment of the color, appearance, texture, odor, or taste of cheese with herbs. In conclusion, wild garlic leaves can be recommended as an additive in the production of soft sheep’s milk rennet-curd cheese.

## Figures and Tables

**Table 1:** Basic chemical composition, acidity, and water activity of sheep cheeses (mean ± SD).

Feature	Type of Cheese and Storage Duration (Weeks)				*p*-Value	
	N		H			
	0	2	0	2	A	T
Water content [%]	53.55 ± 5.25	54.38 ± 1.45	49.67 ± 2.91	50.38 ± 0.82	**	NS
pH	4.58 ± 0.25	4.82 ± 0.03	4.64 ± 0.20	4.72 ± 0.19	*	*
Water activity	0.971 ± 0.002	0.976 ± 0.004	0.966 ± 0.006	0.971 ± 0.005	NS	*
Fat content [%]	23.42 ± 0.92	ne	23.17 ± 3.11	ne	NS	ne
Protein content [%]	17.95 ± 1.35	ne	19.13 ± 1.43	ne	NS	ne
Ash content [%]	2.49 ± 0.22	ne	2.76 ± 0.22	ne	NS	ne
NaCl content [%]	0.94 ± 0.05	ne	0.89 ± 0.05	ne	NS	ne

Abbreviations: N—natural; H—with wild garlic; A—herbal addition effect; T—storage duration effect. * *p* ≤ 0.05; ** *p* ≤ 0.01; NS: *p* > 0.05; ne—not examined. No significant interactions were determined (*p* > 0.05). Values are expressed as the mean of six determinations (two batches × three repetitions) ± the standard deviation (SD).

**Table 2:** Sensory quality of sheep cheeses (mean ± SD).

Feature	Type of Cheese and Storage Duration (Weeks)				*p*-Value	
	N		H			
	0	2	0	2	A	T
Sensory quality on a five-point scale						
Color	4.93 ± 0.24	4.73 ± 0.53	4.98 ± 0.11	4.73 ± 0.53	NS	*
Appearance	4.75 ± 0.41	4.60 ± 0.48	4.58 ± 0.49	4.48 ± 0.57	NS	NS
Texture	4.85 ± 0.33	4.75 ± 0.38	4.63 ± 0.46	4.78 ± 0.34	NS	NS
Odor	4.85 ± 0.33	4.65 ± 0.46	4.93 ± 0.24	4.68 ± 0.44	NS	**
Taste	4.80 ± 0.41	4.60 ± 0.58	4.80 ± 0.41	4.68 ± 0.57	NS	NS
Overall quality	4.83 ± 0.21	4.65 ± 0.36	4.79 ± 0.24	4.66 ± 0.32	NS	*
Intensity of the discriminants of odor and taste						
Sheep milk odor	4.00 ± 0.92	4.05 ± 1.00	2.85 ± 1.23	2.80 ± 1.61	***	NS
Sour odor	2.15 ± 1.31	1.95 ± 1.28	1.95 ± 1.23	1.05 ± 1.00	*	*
Wild garlic odor	0.00 ± 0.00	0.00 ± 0.00	4.30 ± 0.73	4.20 ± 1.01	***	NS
Desirable odor	4.55 ± 0.83	4.35 ± 0.75	4.50 ± 0.76	4.30 ± 0.80	NS	NS
Rancid odor	0.00 ± 0.00	0.30 ± 0.66	0.00 ± 0.00	0.15 ± 0.37	NS	**
Foreign odor	0.00 ± 0.00	0.25 ± 0.55	0.20 ± 0.41	0.20 ± 0.41	NS	NS
Sheep milk taste	4.15 ± 0.99	4.20 ± 0.89	3.30 ± 1.17	3.20 ± 1.47	***	NS
Sour taste	2.65 ± 1.23	2.30 ± 1.22	2.10 ± 0.97	1.90 ± 1.07	NS	NS
Bitter taste	0.10 ± 0.31	0.75 ± 0.97	0.25 ± 0.44	0.75 ± 1.12	NS	*
Piquant taste	0.60 ± 1.14	0.25 ± 0.44	1.60 ± 1.47	1.15 ± 1.39	***	NS
Salty taste	2.90 ± 1.17	2.75 ± 1.29	2.70 ± 1.03	2.25 ± 1.12	NS	NS
Wild garlic taste	0.00 ± 0.00	0.00 ± 0.00	4.00 ± 0.92	4.20 ± 0.70	***	NS
Desirable taste	4.55 ± 0.60	4.40 ± 0.88	4.60 ± 0.60	4.15 ± 1.35	NS	NS
Rancid taste	0.00 ± 0.00	0.10 ± 0.31	0.05 ± 0.22	0.10 ± 0.31	NS	NS
Foreign taste	0.00 ± 0.00	0.05 ± 0.22	0.35 ± 0.67	0.40 ± 0.68	***	NS

Abbreviations: N—natural; H—with wild garlic; A—herbal addition effect; T—storage duration effect. * *p* ≤ 0.05; ** *p* ≤ 0.01; *** *p* ≤ 0.001; NS: *p* > 0.05. No significant interactions were found between effects (*p* > 0.05). Values are expressed as the mean of twenty determinations (two batches × ten panelists) ± the standard deviation (SD).

**Table 3:** Volatile organic compounds in fresh and stored sheep’s milk cheese samples determined by electronic nose with D > 0.9500.

Compounds	Sensory Descriptors ^1^	Type of Cheese and Storage Duration (Weeks)			
		N		H	
		0	2	0	2
1,2-Propanediol, 1-phenyl-	Plastic, pungent, buttery, honey		+		
11-Methyldodecanol	-		+	+	+
1-Decanol, 2-hexyl-	Mild and sweet	+	+	+	+
1-Decanol, 2-methyl-	Sweet and fat-like	+			
1-Decene, 2,4-dimethyl-	-	+			
1-Dodecanol	Fatty and waxy	+			
1-Dodecanol, 2-hexyl-	Fatty and waxy			+	
1-Dodecanol, 2-octyl-	Faint			+	
1-Dodecene	Mild and pleasant	+	+	+	
1-Octanol, 2-butyl-	Pungent, mild, sweet, fishy	+	+	+	+
1-Pentadecene	-				+
1-Tetradecanol	Coconut, weak oily, fatty				+
1-Tetradecene	Mild and pleasant	+		+	
2,4-Dimethyl-1-heptene	Pungent and plastic	+	+	+	+
2,4-Di-tert-butylphenol	Phenolic and herbal	+	+	+	+
2-Decenal, (E)-	Fatty, green, orange, tallowy			+	+
2-Isopropyl-5-methyl-1-heptanol	-	+	+	+	+
3-Ethyl-3-methylheptane	Coffee	+		+	
3-Octadecene, (E)-	-	+			
4-Decene, 7-methyl-, (E)-	Creamy		+		
Acetoin	Butter, coffee, creamy	+	+	+	+
Benzaldehyde	Almond, burnt sugar, fruity, woody	+	+	+	+
Benzene, 1,3-bis(1,1-dimethylethyl)-	-	+	+	+	+
Benzyl alcohol	Aromatic, floral, fruity, sweet	+	+	+	+
Carbonic acid, eicosyl vinyl ester	Sweet		+		
Cyclopentane, 1-butyl-2-propyl-	-		+		+
Decane	Alkane, fruity, fuel, sweet	+	+	+	+
Decane, 4-methyl-	Pungent, acrid, green and ripe fruits, dried pericarp of Japanese pepper	+		+	+
Decane, 5-ethyl-5-methyl-	-	+	+	+	
Dichloroacetic acid, undecyl ester	Pungent and acetic		+		
Dodecane	Alkane and fuel-like	+	+	+	+
Dodecane, 4,6-dimethyl-	-	+	+	+	+
Ethane, 1,1,2,2-tetrachloro-	Sweet			+	
Ethanol, 2-(dodecyloxy)-	-		+		+
Heptane, 2,4-dimethyl-	Pungent and plastic	+	+	+	
Heptane, 4-methyl-	Sweet and fruity	+	+	+	+
Hexadecane	Alkane, fruity, fuel, sweet	+	+	+	+
n-Hexadecanoic acid	Rancid and pungent	+	+	+	+
Nonane, 2,6-dimethyl-	-		+		
Nonane, 4-methyl-	Meaty and fatty	+	+	+	
Octane, 3,3-dimethyl-	-		+		
Octane, 3,5-dimethyl-	Acidic and pungent	+			
Octane, 4-methyl-	Faint hydrocarbon swell		+	+	
Tetradecane	Alkane, fuel, mild herbaceous, sweet	+	+	+	+
Tetradecanoic acid	Waxy, fatty, soapy			+	+
Tetrachloroethylene	Chloroform, sweet, ether-like	+	+		
Undecane, 3,8-dimethyl-	Gasoline-like to odorless	+			

^1^ Sensory descriptors are from the AroChemBase database (Alpha M.O.S., Toulouse, France) or The Good Scents Company Information System. Abbreviations: N—natural; H—with wild garlic; 0—fresh cheese; 2—cheese stored for 2 weeks. “+” means that the compound was detected.

**Table 4:** Color and textural parameters of sheep cheeses (mean ± SD).

Feature	Type of Cheese and Storage Duration				*p*-Value	
	N		H			
	0	2	0	2	A	T
Color parameters						
L*	81.69 ± 1.39	82.06 ± 1.93	75.83 ± 5.96	75.91 ± 5.24	***	NS
a*	−1.85 ± 0.24	−1.96 ± 0.19	−2.38 ± 0.32	−2.52 ± 0.25	***	*
b*	11.76 ± 0.89	13.06 ± 1.51	12.86 ± 1.20	13.87 ± 1.12	***	***
h	98.91 ± 0.67	89.04 ± 9.52	100.57 ± 1.79	91.14 ± 9.51	NS	***
C*	11.91 ± 0.91	7.78 ± 0.75	13.09 ± 1.17	8.20 ± 0.55	***	***
Textural parameters						
Hardness [kG]	1.29 ± 0.26	1.58 ± 0.34	2.16 ± 0.44	2.13 ± 0.39	***	NS
Adhesiveness [kG s]	0.10 ± 0.08	0.13 ± 0.07	0.10 ± 0.09	0.13 ± 0.07	NS	NS
Springiness [-]	0.61 ± 0.07	0.50 ± 0.07	0.63 ± 0.09	0.45 ± 0.08	NS	***
Cohesiveness [-]	0.23 ± 0.04	0.19 ± 0.03	0.23 ± 0.03	0.19 ± 0.02	NS	***
Chewiness [kG]	0.19 ± 0.08	0.15 ± 0.03	0.31 ± 0.11	0.18 ± 0.04	***	***

Abbreviations: N—natural; H—with wild garlic; A—herbal addition effect; T—storage duration effect. * *p* ≤ 0.05; *** *p* ≤ 0.001; NS: *p* > 0.05. Significant interactions (*p* ≤ 0.05) for saturation (C*) and chewiness were determined. Values of color parameters are expressed as the mean of twenty-four determinations (two batches × six cubes × two repetitions) ± the standard deviation (SD). Values of textural parameters are expressed as the mean of twelve determinations (two batches × six cubes) ± standard deviation (SD).

## Data Availability

The data used to support the findings of this study can be made available by the corresponding author upon request.

## References

[1] Balthazar C.F., Pimentel T.C., Ferrão L.L., Almada C.N., Santillo A., Albenzio M., Mollakhalili N., Mortazavian A.M., Nascimento J.S., Silva M.C. (2017). Sheep Milk: Physicochemical Characteristics and Relevance for Functional Food Development. Compr. Rev. Food Sci. Food Saf..

[2] Pluta-Kubica A., Jamróz E., Kawecka A., Juszczak L., Krzyściak P. (2020). Active Edible Furcellaran/Whey Protein Films with Yerba Mate and White Tea Extracts: Preparation, Characterization and Its Application to Fresh Soft Rennet-Curd Cheese. Int. J. Biol. Macromol..

[3] Tarakci Z., Temiz H., Aykut U., Turhan S. (2011). Influence of Wild Garlic on Color, Free Fatty Acids, and Chemical and Sensory Properties of Herby Pickled Cheese. Int. J. Food Prop..

[4] Ptasińska-Marcinkiewicz J. (2014). Hodowla Owiec i Produkcja Mleka Owczego w Polsce i Na Świecie. Zesz. Nauk. Uniw. Ekon. w Krakowie.

[5] Najgebauer- Lejko D., Domagała J., Walczycka M. (2022). Traditional Cheeses from the Malopolska Region. Cultural Heritage—Possibilities for Land-Centered Societal Development.

[6] Sonmezdag A.S. (2019). Characterization of Aroma and Aroma-Active Composition of Gaziantep Cheese by Solvent-Assisted Flavor Evaporation (SAFE) and Aroma Extract Dilution Analysis (AEDA). J. Food Process. Preserv..

[7] Gębczyński P., Bernaś E., Słupski J., Hernik J., Walczycka M., Sankowski E., Harris B.J. (2022). Usage of Wild-Growing Plants as Foodstuff. Cultural Heritage—Possibilities for Land-centered Societal Development.

[8] Znamirowska A., Szajnar K., Rożek P., Kalicka D., Kuzacutẽniar P., Hanus P., Kotula K., Obirek M., Kluz M. (2017). Effect of Addition of Wild Garlic (Allium Ursinum) on the Quality of Kefirs from Sheep’s Milk. Acta Sci. Pol. Technol. Aliment..

[9] Radusin T., Torres-Giner S., Stupar A., Ristic I., Miletic A., Novakovic A., Lagaron J.M. (2019). Preparation, Characterization and Antimicrobial Properties of Electrospun Polylactide Films Containing Allium Ursinum L. Extract. Food Packag. Shelf Life.

[10] Stupar A., Vidović S., Vladić J., Radusin T., Mišan A. (2024). A Sustainable Approach for Enhancing Stability and Bioactivity of Allium Ursinum Extract for Food Additive Applications. Separations.

[11] Sobolewska D., Podolak I., Makowska-Wąs J. (2015). Allium Ursinum: Botanical, Phytochemical and Pharmacological Overview. Phytochem. Rev..

[12] Štajner D., Popović B.M., Čanadanović-Brunet J., Štajner M. (2008). Antioxidant and Scavenger Activities of Allium Ursinum. Fitoterapia.

[13] Pluta-Kubica A., Najgebauer-Lejko D., Domagała J., Štefániková J., Golian J. (2022). The Effect of Cow Breed and Wild Garlic Leaves (*Allium Ursinum* L.) on the Sensory Quality, Volatile Compounds, and Physical Properties of Unripened Soft Rennet-Curd Cheese. Foods.

[14] Gliguem H., Ben Hassine D., Ben Haj Said L., Ben Tekaya I., Rahmani R., Bellagha S. (2021). Supplementation of Double Cream Cheese with Allium Roseum: Effects on Quality Improvement and Shelf-Life Extension. Foods.

[15] Szołtysik M., Dâbrowska A., Babij K., Pokora M., Zambrowicz A., Połomska X., Wojtatowicz M., Chrzanowska J. (2013). Biochemical and Microbiological Changes in Cheese Inoculated with Yarrowia Lipolytica Yeast. Zywn. Nauk. Technol. Jakosc/Food. Sci. Technol. Qual..

[16] (2013). Codex Alimentarius Commission General Standard for Cheese 283-1978.

[17] Znamirowska A., Rożek P., Buniowska M., Kalicka D., Kuźniar P. (2018). Using Wild Garlic (*Allinum Ursinum* L.) in Production of Milk Fermented with Bifidobacterium Animalis Ssp. Lactis BB-12. Zywn. Nauk. Technol. Jakosc Food. Sci. Technol. Qual..

[18] Baryłko-Pikielna N., Matuszewska I. (2014). Rozdział 13. Sensoryczne Badania Konsumenckie—Metody. Sensoryczne Badania Żywności. Podstawy—Metody-Zastosowania.

[19] Naibaho F.G., Hartanto A., Bintang M., Jamilah I., Priyani N., Putra E.D. (2021). GC-MS Analysis and Antimicrobial Activity of the Aqueous Extract from the Bulbs of Allium Chinense G. Don. Cultivated in North Sumatra, Indonesia. Asian J. Agric. Biol..

[20] Dolma N., Shahar B., Chongtham N. (2024). Determination of Mineral Elements, Antioxidant Activity and Bio-Active Compounds of Allium Prezewalskianum Regel, an Underutilized Plant of Ladakh, India Using ICP-AES, AAS and GC-MS. Meas. Food.

[21] Vítová E., Mokáňová R., Babák L., Zemanová J., Sklenářová K. (2011). The Changes of Flavour and Aroma Active Compounds Content during Produgtion of Edam Cheese. Acta Univ. Agric. Silvic. Mendelianae Brun..

[22] Domingues Galli B., Trossolo E., Carafa I., Squara S., Caratti A., Filannino P., Cordero C., Gobbetti M., Di Cagno R. (2024). Effectiveness of Modified Atmosphere and Vacuum Packaging in Preserving the Volatilome of Stelvio PDO Cheese over Time. Food Chem..

[23] Henneberry S., Wilkinson M.G., Kilcawley K.N., Kelly P.M., Guinee T.P. (2015). Interactive Effects of Salt and Fat Reduction on Composition, Rheology and Functional Properties of Mozzarella-Style Cheese. Dairy Sci. Technol..

[24] (2004). Cheese and Processed Cheese—Determination of the Total Solids Content.

[25] (2008). Cheese—Determination of Fat Content—Van Gulik Method.

[26] AOAC (2007). Official Methods of Analysis of AOAC International.

[27] (2017). Foodstuffs—Determination of Water Activity.

[28] Gawęcka J., Jędryka T. (2001). Rozdział 5. Metody Punktowe. Analiza Sensoryczna: Wybrane Metody I Przykłady Zastosowań.

[29] Baryłko-Pikielna N., Matuszewska I. (2014). Rozdział 10. Metody Sensorycznej Analizy Opisowej. Sensoryczne Badania Żywności. Podstawy—Metody—Zastosowania.

[30] Štefániková J., Martišová P., Šnirc M., Kunca V., Árvay J. (2021). The Effect of Amanita Rubescens Pers Developmental Stages on Aroma Profile. J. Fungi.

[31] Najgebauer-Lejko D., Liszka K., Tabaszewska M., Domagała J. (2021). Probiotic Yoghurts with Sea Buckthorn, Elderberry, and Sloe Fruit Purees. Molecules.

[32] Quintanilla P., Beltrán M.C., Molina A., Escriche I., Molina M.P. (2019). Characteristics of Ripened Tronchón Cheese from Raw Goat Milk Containing Legally Admissible Amounts of Antibiotics. J. Dairy Sci..

[33] Domagała J., Pluta-Kubica A., Wieteska-Śliwa I., Duda I. (2022). The Influence of Milk Protein Cross-Linking by Transglutaminase on Technology, Composition and Quality Properties of Gouda-Type Cheese. Int. Dairy J..

[34] XLSTAT Addinsoft (2014). Analyse de Données et Statistique Avec MS Excel.

